# ALDH1B1 Is Crucial for Colon Tumorigenesis by Modulating Wnt/β-Catenin, Notch and PI3K/Akt Signaling Pathways

**DOI:** 10.1371/journal.pone.0121648

**Published:** 2015-05-07

**Authors:** Surendra Singh, John Arcaroli, Ying Chen, David C. Thompson, Wells Messersmith, Antonio Jimeno, Vasilis Vasiliou

**Affiliations:** 1 Department of Pharmaceutical Sciences, University of Colorado Anschutz Medical Campus, Aurora, Colorado, United States of America; 2 Division of Medical Oncology, University of Colorado School of Medicine, Aurora, Colorado, United States of America; 3 Department of Environmental Health Sciences, Yale School of Public Health, New Haven, Connecticut, United States of America; 4 Department of Clinical Pharmacy, University of Colorado Anschutz Medical Campus, Aurora, Colorado, United States of America; University of Dayton, UNITED STATES

## Abstract

In the normal human colon, aldehyde dehydrogenase 1B1 (ALDH1B1) is expressed only at the crypt base, along with stem cells. It is also highly expressed in the human colonic adenocarcinomas. This pattern of expression corresponds closely to that observed for Wnt/β-catenin signaling activity. The present study examines the role of ALDH1B1 in colon tumorigenesis and signalling pathways mediating its effects. In a 3-dimensional spheroid growth model and a nude mouse xenograft tumor model, shRNA-induced suppression of ALDH1B1 expression decreased the number and size of spheroids formed *in vitro* and the size of xenograft tumors formed *in vivo* by SW 480 cells. Six binding elements for Wnt/β-catenin signalling transcription factor binding elements (T-cell factor/ lymphoid enhancing factor) were identified in the human ALDH1B1 gene promoter (3 kb) but shown by dual luciferase reporter assay to not be necessary for ALDH1B1 mRNA expression in colon adenocarcinoma cell lines. We examined Wnt-reporter activity and protein/mRNA expression for Wnt, Notch and PI3K/Akt signaling pathways. Wnt/β-catenin, Notch and PI3K/Akt-signaling pathways were down-regulated in SW 480 cells in which ALDH1B1 expression had been suppressed. In summary, our data demonstrate that ALDH1B1 may promote colon cancer tumorigenesis by modulating the Wnt/β-catenin, Notch and PI3K/Akt signaling pathways. Selective targeting of ALDH1B1 may represent a novel means to prevent or treat colon cancer.

## Introduction

Colorectal cancer is the fourth most commonly diagnosed cancer and second leading cause of cancer related deaths in the United States with 136,830 new cases and 50,310 deaths estimated for year 2014 [1]. Disruptions of several oncogenic signaling pathways have been implicated in colorectal cancer. Of these, mutation-induced constitutive activation of the Wnt/β-catenin pathway is considered to be the most significant [2]. This signaling pathway drives the transformation and tumorigenic progression of colonic epithelial cells [2–4]. Activation of Wnt/β-catenin pathway prevents axin-dependent phosphorylation and degradation of β-catenin [2]. The resultant free β-catenin translocates into the nucleus where it binds and activates T cell factor (TCF)/ lymphoid enhancer factor (LEF) transcription factors [5]. The interaction of the TCF/LEF complex with TCF/LEF binding elements (TBEs) within the promoter results in an increased expression of genes involved in cell proliferation and differentiation, e.g., *c-Myc*, *Cyclin D1* [6]. In the healthy colon, such activation is normally confined to stem or progenitor cells. Other important signaling pathways in colon tumorigenesis include the Notch, phosphoinositide-3-kinase (PI3K), mitogen activated protein kinase (MAPK) and TGFβ signaling pathways [7].

Notch signaling is essential for maintaining normal intestinal homeostasis by influencing cell fate and regulating cell proliferation, differentiation and apoptosis [8]. Dysregulation of Notch signaling has a synergistic effect with Wnt signaling activation that enhances colon cancer development [9,10]. Activation of the Notch pathway modulates transcription of target genes, such as Hairy and enhancer of split (*HES)1*, and *HES5* [11]. Inhibition of Notch signaling causes increased cell differentiation and reduced proliferation in epithelial cells of the intestinal crypt [12]. Conversely, Notch signal activation inhibits differentiation and expands proliferating cells in the intestinal crypt [13]. Jagged1, a Notch ligand, has been shown to be directly controlled by Wnt signaling; hence, the Notch pathway can be indirectly regulated by Wnt/β-catenin signaling [10].

Aldehyde dehydrogenase (ALDH) catalytic activity has been identified as a biomarker for many cancers and cancer stem cells [14]. ALDH1B1 is a relatively unexplored member of the ALDH superfamily. It shares 62% protein identity with ALDH1A1, an ALDH that has garnered much attention recently as a biomarker of cancer stem cells [15]. High Wnt signaling activity is confined to the stem cell compartment of the normal colon and is a distinguishing feature of colon cancer stem cells [16]. We have recently shown that ALDH1B1 expression is localized to stem-like cells at the base of crypts in the normal human colon. In contrast, extremely high ALDH1B1 expression was observed throughout the cells of human colon adenocarcinomas [15]. These results suggest a close association between activation of Wnt/β-catenin signaling and elevated expression of ALDH1B1.

ALDH1B1 metabolizes retinaldehyde to generate retinoic acid (RA) [17], a vitamin A derivative necessary for cell growth and development [14,18]. RA can bind to cellular retinoic acid-binding proteins (CARBPII) and fatty acid binding protein 5 (FABP5), depending on the ratio of FABP5 to CARBPII in the cell [19]. Hence, RA induces CARBPII- or FABP5- mediated activation of retinoic acid receptor (RAR) or PPARβ/δ, respectively. RAR activation induces differentiation and is anti-proliferative whereas PPARβ/δ activation leads to PI3K/Akt-mediated pro-tumorigenic effects. Human colorectal cancer cell lines express very high levels of FABP5 (~30-fold higher than normal colorectal cells), suggesting the possibility of pro-proliferative and anti-apoptotic roles for RA in these cells [19,20]. A role for PI3K/Akt pathway in colon cancer is suggested by the loss of phosphatase and tensin homolog deleted on chromosome 10 (PTEN), a negative regulator of this pathway, in approximately 30% of colorectal cancer cases [21]. The PI3K/Akt signaling pathway could play a crucial role in colon cancer development and maintenance by regulating cell survival, cell cycle progression and cellular growth [22,23]. These findings suggest that, like the Wnt pathway, Notch and PI3K/Akt signaling pathways may also be important for maintaining undifferentiated and proliferative status of colon crypt epithelial cells [24–26].

Given the differential expression pattern of ALDH1B1 in normal and cancerous colon tissues, its apparent correlation with the Wnt/β-catenin pathway [16], and the capacity of ALDH1B1 to modulate RA levels [17], we hypothesize that ALDH1B1 plays an important role in colon tumorigenesis. In the present study, we tested this hypothesis by using human colon cancer cells. We demonstrated that, in addition to acting as a potential colon cancer biomarker, ALDH1B1 may significantly contribute to the development of tumors by modulating canonical Wnt/β-catenin, Notch and PI3K/Akt signaling pathways.

## Materials and Methods

### Cell culture

RKO, SW480, COLO320, HCT116 and HT29 human colon cancer cells (ATCC, Manasss, VA) were cultured in RPMI 1640 medium (Life Technologies, Carlsbad, CA) supplemented with 10% fetal bovine serum (Sigma, Saint Louis, MO), penicillin (100 units/ml), and streptomycin (100 μg/ml) (Life Technologies) at 37°C in a humidified 5% CO_2_ atmosphere.

### Computational analysis of the ALDH1B1 promoter region

The 3-kb promoter region (-1 to -3000) of the human *ALDH1B1* gene (GenBank accession No. NM_000692.4) was analyzed for candidate TBEs using the TESS program.

### Luciferase reporter assay

DNA fragments corresponding to *ALDH1B1* 3 Kb promoter region from nucleotides -3064 to +115 (numbering relative to the transcription start site) were amplified using human genomic DNA (Promega, Madison, WI; G3041). The primers for PCR of the *ALDH1B1* promoter were F (5’-TTAAGGTACCTTCAATCCACACAGGCTCCA-3’) (introduced *Kpn* I as underlined) and R (5’-gttaagctAGCGTTAGTTACCTGCAGCAGG-3’). PCR conditions for this reaction consisted of a denaturing step at 98°C for 30 s and 30 cycles at 98°C for 5 s and 72°C for 50 s. The promoter fragments and pGL3-Enhancer vector (Promega) were digested with KpnI and NheI (New England Biolabs, Ipswich, MA) and ligated into pGL3-Enhancer vector to generate ALDH1B1-pGL3 construct. Using sequential deletion of the putative TBEs from the ALDH1B1-pGL3 construct, four deletion constructs were generated containing: TBE1 to TBE4 (-2381/+115 bp); TBE1 and TBE2 (-1678/+115 bp); TBE1 (-1206/+115 bp) and no TBE (-492/+115 bp). Promoter activity for cMyc was examined using pBV-luciferase vector with cMyc promoter; pBV empty vector was used as a control (Addgene, Cambridge, MA) [27].

SW480 cells were transiently transfected with the reporter constructs or control plasmid (empty pGL3-enhancer or pBV vectors) using FuGENE HD transfection reagent (Promega). Briefly, single cell suspensions of SW480 cells were seeded in a 12-well plate (2.5x10^4^ cells/well) and 2 μg construct was added to each well with 0.2 μg pRL-TK vector (Promega) as an internal control for transfection efficiency. Cells were lysed after 24 h and luciferase activity was measured using Dual-Luciferase Reporter Assay System following the manufacturer’s protocol (Promega). Luminescence was detected using the Synergy 2 plate reader (BioTek, Winooski, VT). This experiment was repeated three times and averaged.

### ALDH1B1 shRNA knockdown

ALDH1B1 shRNA in pRFP-C-RS plasmid vector and pRS vector-negative control (scramble) were purchased from OriGene (Rockville, MD). Stable clones were generated by transfecting SW480 cells in a 6-well plate with 1μg of each of the shRNA plasmids using FuGENE HD transfection reagent (Promega) according to the manufacturer’s recommendations.

### Luciferase reporter assay for Wnt/β-catenin activity

Scramble- or ALDH1B1 shRNA- transfected and non-transfected SW480 cells were plated in 12-well plates to achieve about 50% confluency in 24 h. Since RKO human malignant cells express wild-type APC and β-catenin proteins, we used these cells as a negative control. Cells were then transiently transfected using FuGENE HD transfection reagent (Promega) according to the manufacturer’s protocol with either 2.5 μg TOPflash or FOPflash reporter plasmid (Millipore, Temecula, CA) along with 0.2 μg pRL-TK vector (Promega) as an internal control for transfection efficiency. The TOPflash plasmid contains TCF4 binding sites upstream of the luciferase gene, which is responsive to the presence of active Wnt/β-catenin signaling, whereas the FOPflash plasmid contains mutated TCF4 binding sites. Cells were lysed and luciferase and renilla luminescence were then measured in a 96-well plate using the Dual-Luciferase Reporter Assay System (Promega). The ratio of TOPflash/FOPflash signal was calculated and normalized to non-transfected and scramble sh-RNA transfected cells.

### Immunohistochemical analysis

Colon tissue sections from *Apc* ^*Min*^ mice were kindly provided by Dr. Jeffrey Peters (Pennsylvania State University). These were subjected to immunohistochemical staining combined with tyramide signal amplification system (TSA Biotin System; NEN Life Science Products, Boston, MA) as previously described [28]. Sections were incubated with rabbit polyclonal anti-ALDH1B1 antibody [29] at a dilution of 1:750 in TNB blocking buffer overnight in a humidified chamber. HRP-conjugated anti-rabbit secondary antibody was used at a dilution of 1:500 in TNB blocking buffer for 60 min. Sections were visualized by incubating with DAB working solution (Vector laboratories) for 10 min at room temperature and counterstained with diluted hematoxylin (1:10 with distilled water) for 2 min. Finally, sections were dehydrated in ascending concentrations of ethanol and mounted with Permount (Sigma) for microscopic examination.

### Western blotting

Cells lysates were prepared as described previously [15] and 50 μg of protein was loaded and separated on a 10% SDS polyacrylamide gel. Western blot analysis was performed as previously described using rabbit polyclonal anti-ALDH1B1 antibody (1:5,000 in 5% nonfat dry milk in TBST) [15] and β-actin was detected using mouse monoclonal anti-β-actin antibody (1:10,000; Sigma). Active β-catenin, LEF1, c-Myc, JAG1, c-Notch1, pI3Kp85, Akt and MMP2 were detected using corresponding rabbit polyclonal antibodies (1:1,000; cell signaling technology, Danvers, MA). PPARδ and FABP5 were detected using corresponding rabbit polyclonal antibodies (1:1,000; abcam, Cambridge, MA). HRP-conjugated goat anti-rabbit or anti-mouse secondary antibody (1:5,000; Sigma) were used. Quantitation of band density was conducted using NIH ImageJ software (http://rsbweb.nih.gov/ij/) [30].

### Gene expression

RT-PCR was used to evaluate canonical Wnt/β-catenin pathway genes in human SW480 cells before and after shRNA-mediated ALDH1B1 knockdown. Total RNA was extracted using RNeasy Mini kit (Qiagen, Valencia, CA) followed by cDNA synthesis using Maxima Universal First-Strand cDNA Synthesis kit (Thermo) following manufacturer’s instructions. Validated and pre-designed primer/probes for *Axin2*, *c-Myc*, *LGR5* and *GAPDH* were purchased from Applied Biosystems. AB7500 system (Applied Biosystems, Carlsbad, CA) was used to run RT-PCR using Taqman master mix. Relative quantification of the steady-state target mRNA levels was calculated after normalization of total amount of cDNA to GAPDH (endogenous reference). Relative mRNA expression was analyzed by using the ΔΔ*CT* method, where (Δ*C*_*T*_) = C_T_ (gene)—C_T_ (housekeeping gene).

### Three-dimensional cell cultures

SW480 cells were cultured by the overlay method of 3D culture on solidified Matrigel (BD Biosciences, San Jose, CA) [30]. Briefly, cells were grown to ~ 80% confluency in a monolayer. A single cell suspension was prepared in RPMI 1640 medium supplemented with 10% fetal bovine serum, penicillin (100 units/ml), streptomycin (100 μg/ml) (Life Technologies) and 2% Matrigel and seeded onto 80 μl Matrigel in an 8-well chamber slide (BD Biosciences) at 2000 cells/well. All cultures were then maintained at 37°C in a 5% CO_2_ humidified incubator for up to 10 d. Cell morphology was examined every alternate day and the size of resulting spheroids was measured on day 10 and volume was calculated by formula V = (4/3)πr^3^. Image analysis was done using Zeiss Axioscop 2 microscope (Carl Zeiss, Inc., Germany) and Carl Zeiss Axiovision Rel 4.5 software.

### Ethics statement

The care and the treatment of mice were in accordance with the University of Colorado institutional guidelines, and under a protocol reviewed and approved by the University of Colorado Institutional Animal Care and Use Committee (IACUC) responsible for the regulation of animal research.

### Xenograft in athymic mice

Scramble control and ALDH1B1 shRNA knockdown SW480 cells were injected into the left and right flanks of 4 to 6 wk-old female athymic (nu^+^/nu^+^) mice. These mice were obtained from Harlan Laboratories, (Indianapolis, IN). Mice were monitored daily for signs of toxicity, and the tumor size was evaluated twice/wk by caliper measurement using the following formula: tumor volume = (length × width^2^) × 0.52. Mice were euthanized at the end of the experiment. All protocols used were approved by the Institutional Animal Care and Use Committee of the University of Colorado Denver.

### Flow cytometric analysis of cells

Cell count and viability of scramble and ALDH1B1 shRNA-transfected SW480 cells were determined on a Countess Automated Cell Counter (Life Technologies). Cells in both groups exhibited more than 80% viability. The Aldefluor reagent system (Stem Cell Technologies, Vancouver, Canada) was used to detect intracellular catalytic activity of ALDH. In brief, cells were centrifuged (250 G for 5 min) and pellets were resuspended in Aldefluor assay buffer containing 15 μM BAAA at a concentration of 10^6^ cells/ml. Activated Aldefluor (5 μl) was added to 1 ml of resuspended cells and mixed well. For a negative control, 500 μl from this cell suspension was immediately placed in another tube containing 5 μl ALDH inhibitor [diethylaminobenzaldehyde (DEAB)]. All samples were incubated at 37°C for 45 min and then centrifuged and washed with 500 μl Aldefluor buffer. ALDH^+^ cells were evaluated by the University of Colorado Cancer Center flow cytometry core facility.

### Statistical analysis

All quantitative experiments were performed at least in triplicate, and the data are presented as the means ± S.E. Statistical significance was determined by Student’s unpaired t-test or one-way ANOVA using SigmaStat Statistical Analysis software (SPSS Inc., Chicago, IL). P < 0.05 was considered to be significant.

## Results

### ALDH1B1 expression in colon polyps from *Apc* ^*Min*^ mice

**Fig 1:**
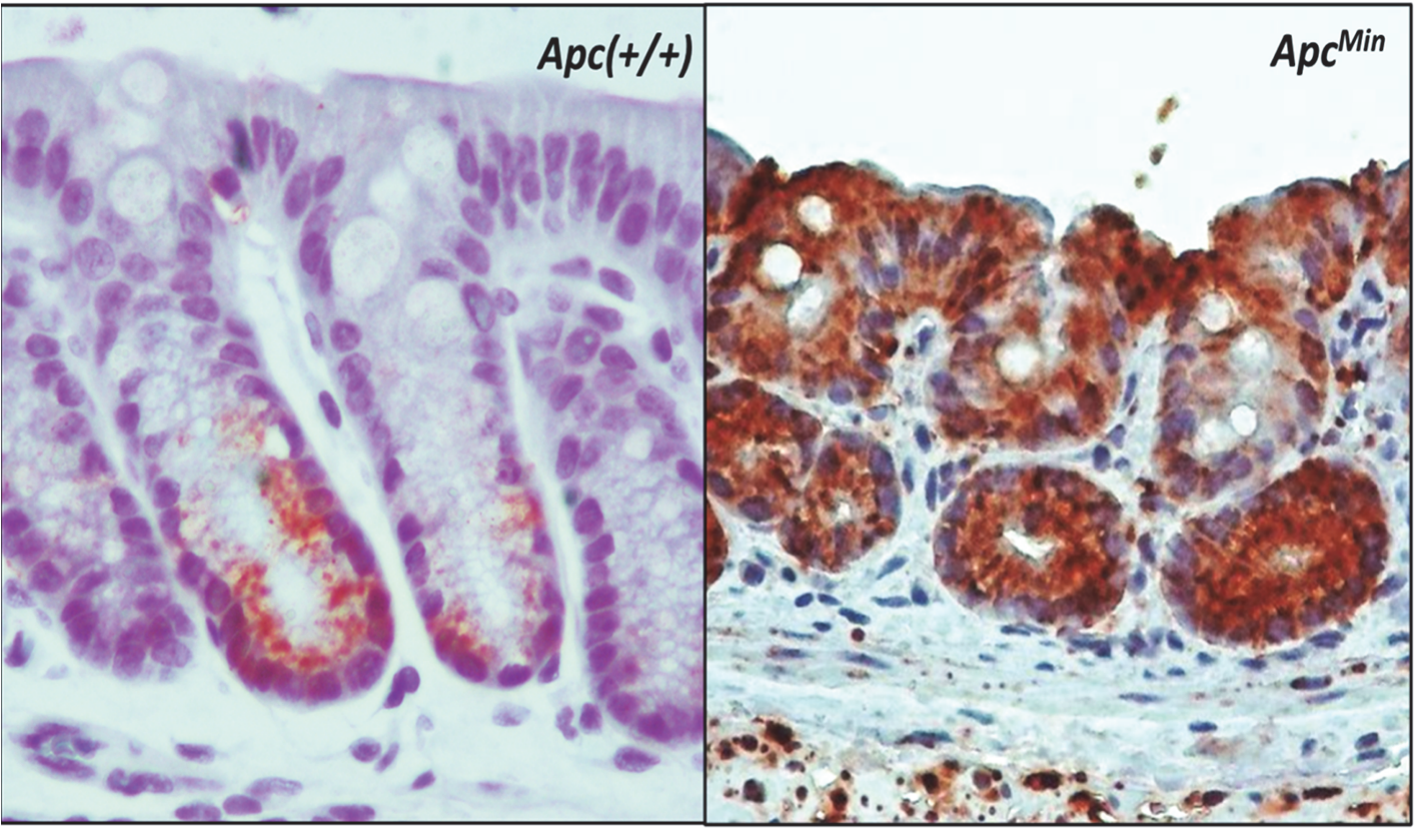
Immunohistochemical localization of ALDH1B1 in the colon of wild-type and *ApcMin* mice. Expression of ALDH1B1 (brown stain) is confined to stem cell region of the normal colon (left panel) from wild-type mice (*Apc(+/+)*). In contrast, a colon polyp from *ApcMin* mice showed universal expression in the epithelial cells, with some staining in the stromal cells (right panel). ALDH1B1 expression exhibited cytosolic punctate pattern. (100x)

The colon from wild-type (*Apc(+/+)*) mice expressed immunopositivity for ALDH1B1 in a small subset of the columnar cells at the bottom of crypt (i.e., in the putative stem cell compartment) and in some cells along the side of the lower middle part of the crypt (Fig 1). However, cells from stroma and differentiated mucosal cells remained completely negative. In contrast, colon polyps from *Apc* ^*Min*^ mice showed very strong and universal ALDH1B1 expression in the epithelial cells. Some of the stromal cells from *Apc* ^*Min*^ mice were also stained positively for ALDH1B1 (Fig 1).

### Identification of novel TBE sites in the promoter of the human ALDH1B1 gene

**Fig 2:**
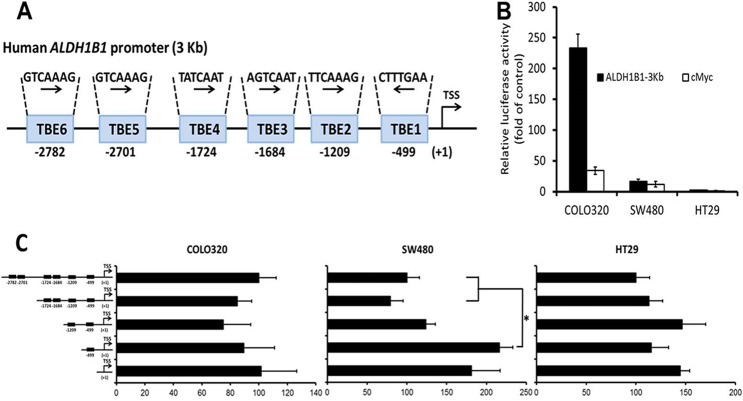
Evaluation of human *ALDH1B1* promoter. (**A**) Putative TCF/LEF binding elements (TBEs) in the human ALDH1B1 3-Kb promoter region. TBE6, TBE5, TBE4, TBE3 and TBE2 are in sense orientation whereas TBE1 is in antisense orientation. Numbers given below TBEs represent the distance from transcription start site (TSS) in base pairs. (**B**) Activity of the 3kb ALDH1B1-pGL3 and cMyc-pBV promoter reporter constructs expressed relative to control (the empty pGL3-enhancer and pBV vectors, respectively) in COLO320, SW480 and HT29 cells. The Firefly luciferase activity is normalized to the internal control Renilla luciferase. Data are presented as mean ± SEM (representative from three individual experiments done in triplicate). (**C**) Activity of the entire 3kb ALDH1B1 pGL3-promoter construct and truncations of the construct in which putative TCF/LEF binding elements (TBEs) have been sequentially deleted. Luciferase promoter activity was measured 24h after transfection and the cells transfected with empty plasmid were used as control. The Firefly luciferase activity is normalized to the internal control Renilla luciferase. Luciferase activity was calculated as fold of empty pGL3 enhancer vector and data are expressed as per cent luciferase activity of TBE1-6 construct and presented as mean ± SEM (representative from three individual experiments done in triplicates). *P < 0.05, one-way ANOVA.

Since ALDH1B1 is overexpressed in human colon cancer and appears to be associated with activation of Wnt/β-catenin pathway, we analyzed the human ALDH1B1 3kb promoter region using the Transcription Element Search System (TESS) program (www.cbil.upenn.edu/cgi-bin/tess/). Six binding sites for TCF/LEF transcription factors were identified in the 3000 bp upstream sequence from transcription start site of the ALDH1B1 gene, which comprise a minimum element of 5′-(A/T)(A/T)CAA(A/T)G-3′ (Fig 2A) [31]. TBE6 (-2782 bp), TBE5 (-2701 bp), TBE4 (-1724 bp), TBE3 (-1684 bp) and TBE2 (-1209 bp) were in sense orientation whereas TBE1 (-499 bp) was in antisense orientation. Therefore, we cloned the 3064 bp segment from the human ALDH1B1 promoter (including all six TBE sites) into the pGL3-enhancer vector (ALDH1B1-pGL3). This vector was used for the subsequent reporter assay (Fig 2B).

### ALDH1B1 promoter is active in colon cancer cells

Investigation of ALDH1B1 gene expression among colon cancer cells showed that the COLO320 cell line had the greatest expression of ALDH1B1 mRNA followed by SW480 and HT29 (Matsumoto et al., manuscript in preparation). Since these cells exhibited high levels of ALDH1B1, we transfected the SW480, COLO320 and HT29 cell lines with ALDH1B1-pGL3 or cMyc-pBV luciferase vector and used corresponding empty plasmids as a control. Luciferase activity was measured to determine the basal level of activation of the promoters for *ALDH1B1* and *c-Myc* in these colon cancer cells. *ALDH1B1* promoter activity was 2-, 17- and 233-fold that of the control plasmid in the HT29, SW480 and COLO320 cells, respectively. A similar cell line order of activity was observed for the *c-Myc* promoter activity (Fig 2B). These results suggest that the *ALDH1B1* promoter activation pattern in colon cancer cells closely resembles that of *ALDH1B1* mRNA expression and *c-Myc* promoter activity in these cells. To dissect the contribution of Wnt/β-catenin-responsive TBE sites on the transcriptional activity of the *ALDH1B1* promoter, four *ALDH1B1* promoter deletion constructs were assayed for their activity through transient transfections in COLO320, SW480 and HT29 cells. Surprisingly, sequential deletion of all of the putative TBEs from the ALDH1B1-pGL3 construct did not influence the promoter activity in COLO320 and HT29 cells whereas deletion of TBE2 to TBE6 in SW480 cells led to increased luciferase activity (Fig 2C). We also examined the remaining promoter region (-492/+115 bp) after deletion of the six TBEs for the presence of binding sites of other transcription factors, such as AP-2, C2H2 zinc finger, E2F4, EGR1, ETS1, and serum response factors (data not shown), several of which are known to play an important role in colon cancer.

### ALDH1B1 plays a critical role in colon cancer tumorigenesis

**Fig 3:**
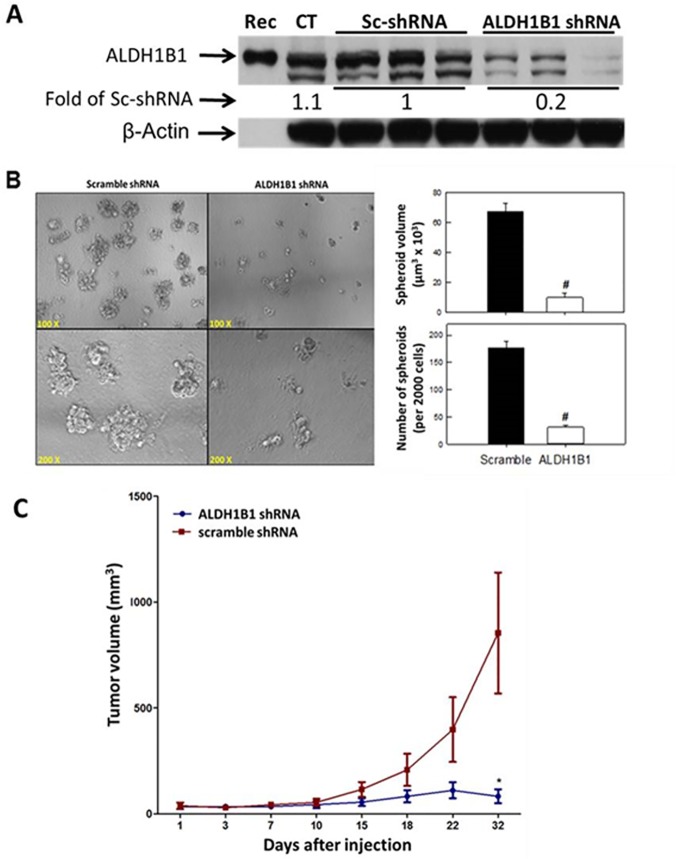
Decreased ALDH1B1 expression inhibits spheroid formation and tumor growth. SW480 cells were transfected with ALDH1B1 shRNA or scramble shRNA to develop stable cell colonies. (**A**) ALDH1B1 expression was examined by Western blotting and quantified by densitometric analysis. Numbers at the bottom of ALDH1B1 Western blot bands are the average densitometry of three replicates expressed relative to scramble shRNA-transfected (control) cells. Rec, recombinant ALDH1B1; CT, Control SW480 cells without transfection. (**B**) Spheroid formation was measured 10 d after plating of cells. Representative phase contrast micrographs (left) and histograms (right) showing the number and volume of spheroids developing from SW480 cells transfected with ALDH1B1 shRNA (open bars) or with scramble shRNA (control) (closed bars) vectors. Data are presented as mean ± SEM (n = 3). # *P* < 0.01, Student’s unpaired t-test, compared with scramble shRNA. (C) Growth of tumors induced by injection of SW480 cells transfected with ALDH1B1 shRNA (circles) or scramble shRNA (squares)) into the left and right flanks of female athymic (nu+/nu+) mice. Tumor volume was calculated as (length × width^2^ × 0.52). Data are presented as mean ± SEM (n = 10 tumors per group). * *P* < 0.05, Student’s unpaired t-test, compared with tumors in scramble shRNA group at same time point.

We used the SW480 cell line to examine the effect of suppressing ALDH1B1 expression on tumor growth. This was accomplished by shRNA-mediated knockdown. The effectiveness of shRNA knockdown was evaluated by Western blotting and ALDH1B1 expression was decreased in the ALDH1B1 shRNA cells by 80% (Fig 3A).

To evaluate the effect of ALDH1B1 knockdown on tumor growth, we used an *in vitro* three-dimensional culture system and an *in vivo* xenograft model. Cells from the scramble (control) and ALDH1B1 knockdown groups were grown in three-dimensional culture to examine spheroid formation. After 10 d of incubation, the volume and number of spheroids were lower in the ALDH1B1 knockdown cell cultures than in control cell cultures (Fig 3B). Furthermore, tumor volume in a xenograft model was reduced in tumors derived from ALDH1B1 knockdown cells than in those derived from scramble control cells (Fig 3C).

### ALDH1B1 knockdown depletes ALDH^bright^ cells

**Fig 4:**
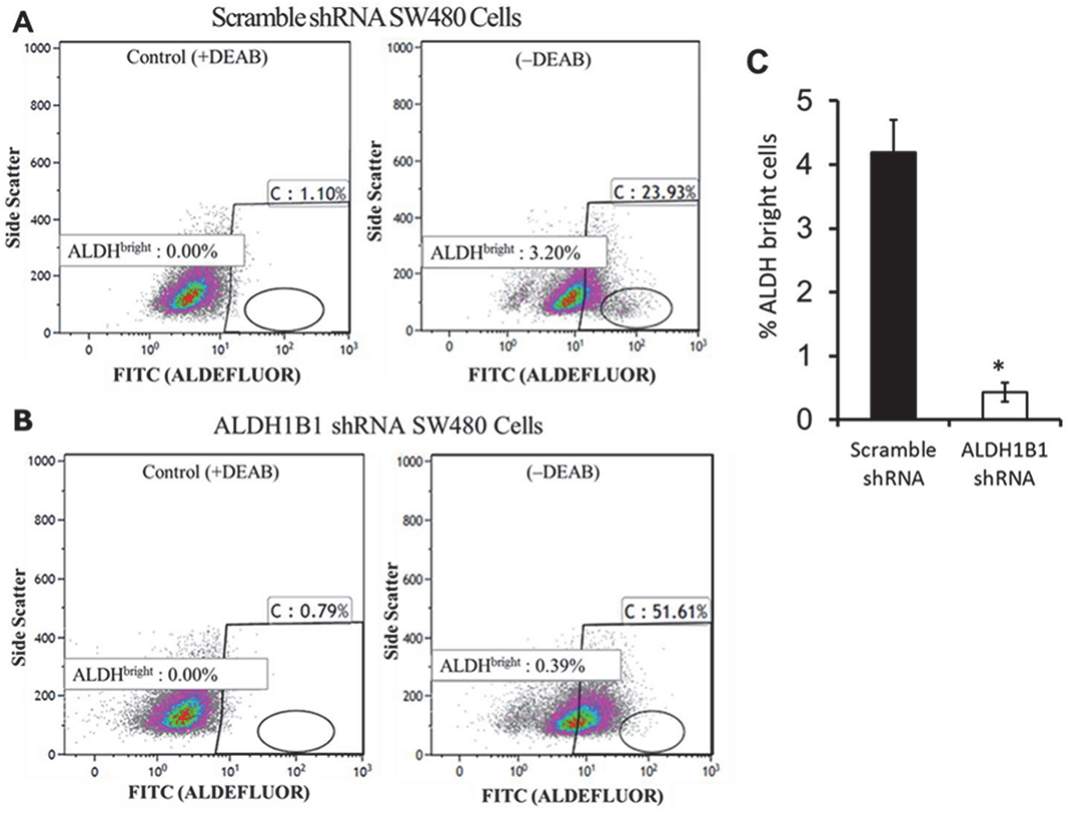
ALDH1B1 knock down depletes ALDH^bright^ cells. Flow cytometry was conducted in SW480 cells transfected with either ALDH1B1 shRNA or scramble shRNA. Cells were incubated with Aldefluor substrate in the presence or absence of the ALDH inhibitor diethylaminobenzaldehyde (DEAB). Cells with high fluorescence (reflecting high ALDH activity, i.e., ALDH^bright^ cells) were determined and arbitrarily defined as being in the oval gate in the bottom right quadrant of the graph. (**A**) Flow cytometric analysis of scramble shRNA control cells in the absence (-DEAB) or presence (+DEAB) of DEAB. (**B**) Flow cytometric analysis of ALDH1B1 shRNA cells in the absence (-DEAB) or presence (+DEAB) of DEAB; (**C**) Proportion of ALDH^bright^ cells in ALDH1B1 shRNA (black bar) and scramble shRNA (white bar) cell populations. Data are presented as the mean ± SEM from 3 experiments. * *P* < 0.05, Student’s unpaired t-test, compared with scramble shRNA cells.

Scramble shRNA transfected (control) SW480 cells contain a small population of cells (~ 3%) with very high ALDH catalytic activity, so-called ALDH^bright^ cells (Fig 4A). The capacity of DEAB treatment to abolish ALDH^bright^ cells (Fig 4A) is consistent with their fluorescence being associated with ALDH catalytic activity. ALDH1B1 knockdown in SW480 cells using shRNA decreased the number of ALDH^bright^ cells by about 90% (Fig 4B and 4C). Our findings suggest that ALDH1B1 may be the ALDH isozyme responsible for the catalytic activity of the ALDH^bright^ population of cells in the present study.

### ALDH1B1 regulates Wnt/β-catenin, Notch and PI3K/Akt signaling pathways in colon cancer

**Fig 5:**
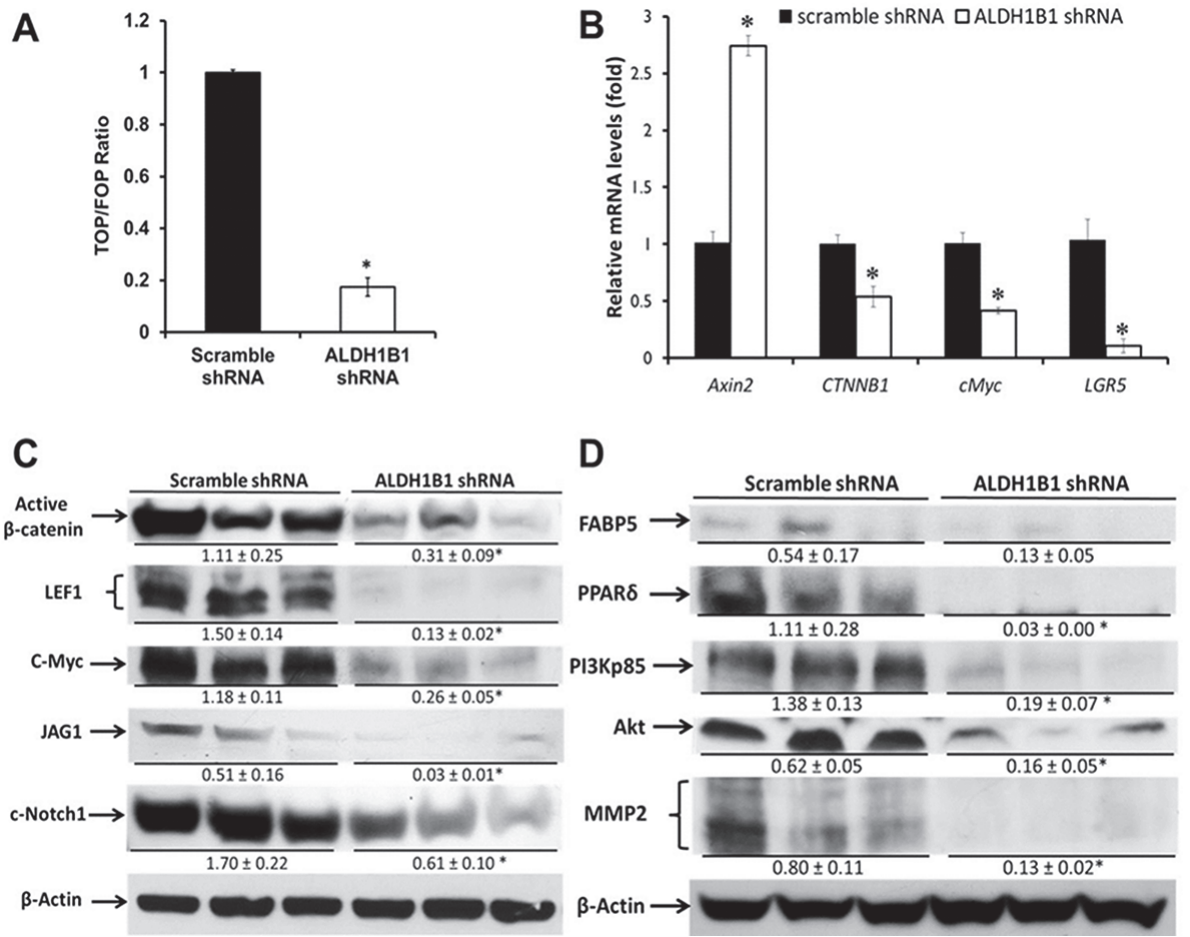
ALDH1B1 modulates Wnt/β-catenin, Notch and PI3K/Akt signaling pathways. (**A**) For the Wnt/β-catenin reporter assay, scramble or ALDH1B1 shRNA transfected SW480 cells were transiently transfected with either TOPflash, or FOPflash reporter plasmid along with pRL-TK vector as an internal control. Data are expressed as TOP/FOPflash ratio and presented as mean ± SEM (representative from three individual experiments done in triplicate). * *P* < 0.05, Student’s unpaired t-test. (**B**) RT-PCR analysis of RNA isolated from SW480 cells transfected with scramble shRNA or ALDH1B1 shRNA. mRNA levels were expressed as a ratio of the levels in scramble shRNA-transfected cells. *GAPDH* gene expression was used to normalize data. Results are presented as the mean ± SEM from three experiments. * *P < 0*.*05*, Student’s unpaired t-test, compared with results in scramble shRNA cells. Western blot analysis of expression of members of Wnt/β-catenin and Notch pathways (**C**) and of FABP5, PPARβ/δ and PI3K/Akt pathway members (**D**) were conducted in ALDH1B1 shRNA and scramble shRNA cells. Western blots were quantified by densitometry, numbers represent mean ± SEM of three independent experiments. * *P* < 0.05, compared to results in scramble shRNA cells.

Because Wnt-signaling activation and ALDH1B1 expression appear to be closely linked in human colon cancer and *Apc* ^*Min*^ mice, we examined the effect of ALDH1B1 knockdown on Wnt/β-catenin signaling. We employed the TOPFlash/FOPFlash Wnt activity reporter assay using scramble (control) shRNA and ALDH1B1 knockdown SW480 cells. Decreased β-catenin-mediated transcriptional activity occurred in SW480 ALDH1B1 knockdown cells 24 h after shRNA transfection (Fig 5A).

We then examined the expression levels of the Wnt/β-catenin signaling-related genes *Axin2*, *CTNNB1* (β-catenin), *c-Myc* and *LGR5*. RT-PCR analysis showed a strong induction of *Axin2* and suppression of *CTNNB1*, *c-Myc* and *LGR5* expression in the ALDH1B1 knockdown SW480 cells (Fig 5B). ALDH1B1 knockdown decreased expression of active β-catenin, LEF1 (crucial proteins for Wnt/ β-catenin signaling) and c-Myc (Wnt target) (Fig 5C). Since JAG1, the Notch ligand, is regulated by Wnt-signaling, we investigated the effects of ALDH1B1 knockdown on JAG1 protein levels as well as on cleaved Notch1 expression. As shown in Fig 5C, both JAG1 and cleaved Notch1 were decreased in the SW480 ALDH1B1 knockdown cells. Taken together, these findings indicate that ALDH1B1 positively modulates Wnt/β-catenin and Notch signaling pathways in SW480 colon cancer cells.

ALDH1B1 can be involved in the generation of RA [17] and, in cells expressing high FABP5, RA plays a pro-proliferative role by modulating PPARβ/δ activity. PPARβ/δ has been shown to enhance the activity of PI3K/Akt signaling [19]. Therefore, we examined the expression of FABP5, PPARβ/δ and PI3K/Akt pathway-related proteins, specifically PI3K-p85, Akt and MMP2. Knockdown of ALDH1B1 in the SW480 cells resulted in the down-regulation of all of these proteins (Fig 5D).

## Discussion

Despite a better understanding and substantial advances in our knowledge of the molecular mechanisms involved in colorectal cancer development and progression, this disease still remains one of the leading causes of cancer-related deaths in the developed world. Colorectal cancers show dysregulation of the Wnt/β-catenin signaling pathway, including Wnt-dependent transcription of *c-Myc* and *CyclinD1*. Our previous work showed that ALDH1B1 is a potential biomarker for colon cancer and the expression pattern of this protein is suggestive of a close association of ALDH1B1 with the activation of Wnt/β-catenin signaling, a crucial pathway for colon cancer generation.

High levels of ALDH1B1 in human colon cancer cells are suggestive of a role for ALDH1B1 in colon tumorigenesis. To examine this possibility, we used an shRNA approach to suppress or knock down expression of ALDH1B1 in SW480 cells, a human colon cancer cell line. The shRNA transfection caused an 80% reduction in ALDH1B1 levels. We selected SW480 cells for tumor formation assays because they exhibit high constitutive expression of ALDH1B1 and very low constitutive ALDH1A1 expression (Matsumoto et al., manuscript in preparation). Hence, any possible obfuscating effects of ALDH1A1 on tumorigenicity would be minimized. Suppression of ALDH1B1 expression by shRNA reduced spheroid formation by SW480 cells in a three-dimensional cancer cell culture and reduced tumor growth by SW480 cells in an *in vivo* xenograft model. On the basis of our findings, it may be proposed that ALDH1B1 has a pro-tumorigenic role in human colon cancer cells. This may explain its widespread expression in human colon cancers. These results are consistent with ALDH1B1 having a pro-tumorigenic role in human colon cancer cells. This is contrary to the majority of previous studies which have suggested ALDH1A1 as being the isozyme primarily responsible for the ALDH catalytic activity observed in cancer cells [32–38]. However, we and others have shown that other ALDHs may contribute to the catalytic activity measured by the Aldefluor assay [15,39]. Cells with very high ALDH catalytic activity, so-called ALDH^bright^ cells, constitute a small subpopulation of tumor cells with stem cell-like properties (stemness), including high tumorigenicity, self-renewal and chemoresistance [32,40]. Knockdown of ALDH1B1 greatly reduced the prevalence of ALDH^bright^ cells, showing that ALDH1B1 was responsible for the high ALDH activity of ALDH^bright^ SW480 colon cancer cells. Given that ALDH^bright^ cells are considered to possess high tumorigenic potential [41], the results are consistent with ALDH1B1 playing an important role in tumorigenesis of colon cancer cells.

Several inherited and sporadic colorectal cancer studies have established that Wnt/β-catenin signaling plays a critical role in colon tumorigenesis [2]. With our discovery that ALDH1B1 may be central for colon cancer formation and is a major contributor of ALDH activity in highly tumorigenic ALDH^bright^ SW480 colon cancer cells, we examined the relationship between ALDH1B1 and the Wnt/β-catenin signaling pathway. First, we hypothesized that ALDH1B1 may be regulated by Wnt/β-catenin signaling. Since activation of Wnt-signaling is mediated through binding of β-catenin to TCF/LEF transcription factors, the presence of TBE is required in the promoter regions of all the genes under direct control of the Wnt/β-catenin signaling pathway [42–44]. In this study, we found six potential TBE sites to be present in the *ALDH1B1* 3kb promoter region, making it a potential target for control by β-catenin and, by extension, the Wnt/β-catenin signaling pathway [2]. In addition, we demonstrated that the *ALDH1B1* promoter was transcriptionally active in colon cancer cell lines. However, even after sequential deletion of all six TBE sites from the ALDH1B1-pGL3 promoter construct, promoter activity remained the same. This indicates that none of the potential TBE sites found within the 3 kb promoter region of *ALDH1B1* are contributing to the transcriptional activation of the ALDH1B1 promoter. In SW480 cells, deletion of the promoter region containing TBE2 to TBE6 resulted in increased ALDH1B1 promoter activity, suggesting that transcription repression factors are present in this region of the promoter and are functional in these cells. Given that all of the examined cell lines showed ALDH1B1 promoter activity even after deletion of all the six putative TBEs, it may be speculated that the remaining promoter region (-492/+115 bp) has some binding elements for transcriptional activators. We explored this possibility and found binding sites for various transcription factors known to be important in colon cancer [45–50]. Further research is required to ascertain that which of these sites is important in promoting ALDH1B1 expression. Next, we hypothesized that ALDH1B1 affected colon cancer formation by modulating Wnt/β-catenin signaling in colon cancer cells. In the present study, knockdown of ALDH1B1 expression in SW480 cells was associated with increased expression of *Axin2* mRNA and suppression of *CTNNB1*, *c-Myc* and *LGR5* mRNAs. *Axin2* negatively regulates Wnt/β-catenin signaling and mutations in *Axin2* have been described to be oncogenic in colorectal cancer [51,52]. *C-Myc* is an oncogene and downstream target of Wnt/β-catenin signaling [2,53] and overexpression of c-Myc has been shown to enhance cell proliferation and survival in cancer cells [54]. Downregulation of c-Myc suppresses growth in colon cancer cells and induces apoptosis [55]. LGR5 is a target gene of Wnt/β-catenin signaling and is expressed in stem-like cells in colorectal cancers. In addition, overexpression of LGR5 is associated with a poor prognosis in colorectal cancer patients [53,56]. Collectively, these findings suggest that ALDH1B1 may modulate colorectal cancer formation through a mechanism involving the Wnt/β-catenin signaling pathway.

Overexpression of Notch-related proteins in cells with active Wnt signaling at the bottom of intestinal crypt is suggestive of cross-talk between Wnt and Notch signaling [13,26]. In addition *Apc* ^*Min*^ mice, which have mutated *Apc* gene and resultant hyperactive Wnt signaling, show enhanced Notch signaling activity [57]. Overexpression of the Notch signaling pathway has been shown to dramatically increase proliferating cells in wild-type mice. However, a similar effect is not seen in TCF-4 null mice, which have blocked Wnt activity [58]. This indicates that expansion of proliferative compartments in response to hyperactivation of Notch signaling requires an intact Wnt-signaling cascade [58]. The synergistic effect of these two pathways on tumor growth has also been shown in *Notch*/*Apc* ^*Min*^ double mutant mice. These mice exhibited an increase in the number of intestinal tumors and dysplastic lesions in the colon relative to the *Apc* ^*Min*^ mice [58]. In addition, the Notch ligand Jagged1 is transcriptionally activated by β-catenin, suggesting Notch signaling is downstream of the Wnt pathway in colorectal cancer [10]. Our results showing down-regulation of the Notch pathway along with Wnt signaling down-regulation in ALDH1B1 knockdown cells are consistent with these results. These findings, when taken together with the enhanced Notch activity identified in human colon adenoma [58], suggest that Notch signaling may enhance tumor development by hyper-activation of Wnt signaling.

**Fig 6:**
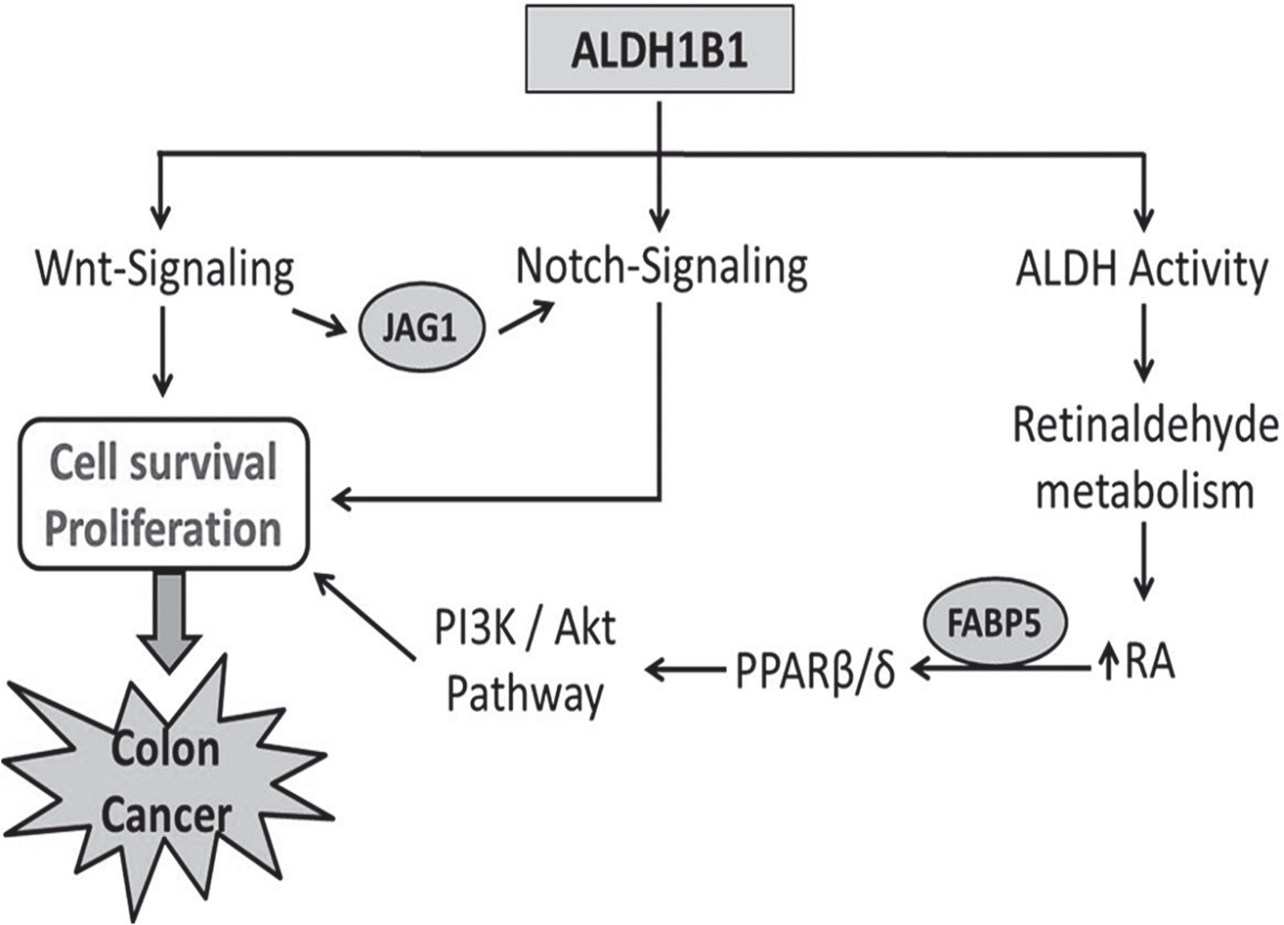
Proposed mechanism by which ALDH1B1 modulates colon tumorigenesis. ALDH1B1 play important role in cell survival and proliferation by modulating Wnt-, Notch- and PI3K/Akt signaling pathways and thereby contributes in colon tumorigenesis.

The mechanism by which ALDH activity promotes tumor growth is the subject of intensive research. Retinoic acid (RA) has been reported to increase expression of pro-survival genes and tumor growth by activating PPARβ/δ in colon cancer in cells with high FABP5 relative to CARBPII, which in turn can modulate PI3K/Akt pathway [19,59]. This may explain the decrease in tumorigenicity observed in the ALDH1B1 knockdown cells in our *in vitro* and *in vivo* tumor formation assays. Our results show that PI3K/Akt pathway is down-regulated upon ALDH1B1 suppression, possibly by down-regulating PPARβ/δ which corroborates earlier findings and suggests an association between this pathway and colorectal cancers [23]. A summary of the mechanisms by which we believe ALDH1B1 can modulate colon cancer is provided in Fig 6.

Our findings demonstrate that ALDH1B1 may not only be a potential biomarker for colon cancer but also play a functional role in the formation of tumors in colorectal cancer. In addition, the present study provides mechanistic insights into the means by which ALDH1B1 may promote tumor formation, i.e., by modulating the Wnt/β-catenin, Notch, and PI3K/Akt signaling pathways. Finally, it is apparent that ALDH1B1 can be a significant contributor to ALDH activity measured by the Aldefluor assay. These results substantiate the accumulating evidence that ALDH isozymes other than ALDH1A1 may contribute to the cancer stem cell phenotype identified by the Aldefluor assay [15]. As such, ALDH1B1 may be one of the factors imparting high tumorigenicity to these tumor-initiating cells in colon cancer. It is anticipated that the findings from this study will serve as a foundation that may allow earlier detection of colorectal cancer and the identification of novel therapeutic approaches for the prevention and treatment of this devastating disease, such as specific inhibitors or substrates of ALDH1B1.
